# Characteristics of persons who died by suicide in prison in France: 2017–2018

**DOI:** 10.1186/s12888-021-03653-w

**Published:** 2022-01-04

**Authors:** Alexis Vanhaesebrouck, Amélie Tostivint, Thomas Lefèvre, Maria Melchior, Imane Khireddine-Medouni, Christine Chan Chee

**Affiliations:** 1 Interdisciplinary Research Institute On Social Issues (IRIS), UFR SMBH, Université Sorbonne Paris Nord, UMR 8156-997 Paris, France; 2 Department of Legal and Social Medicine, Hôpital Jean-Verdier (AP-HP), 93140 Bondy, France; 3 Pierre Louis Institute of Epidemiology and Public Health, Department of Social Epidemiology, Sorbonne Université, INSERM, Paris, France; 4 Health division of the National Prison Service, Ministry of Justice, Paris, France; 5 National Agency of Public Health (Santé Publique France), Saint-Maurice, France

**Keywords:** Suicide, Prison, Social psychiatry, Social epidemiology

## Abstract

**Background:**

In northern countries, suicide rates among prisoners are at least three times higher for men and nine times higher for women than in the general population. The objective of this study is to describe the sociodemographic, penal, health characteristics and circumstances of suicide of French prisoners who died by suicide.

**Methods:**

This study is an intermediate analysis of the French epidemiological surveillance program of suicides in prison. All suicides in prison in 2017–2018 in France were included in the study. Archival sociodemographic and penal data and specific data on the circumstances of the suicidal act were provided by the National Prison Service. Health data was provided by physicians working in prison using a standardized questionnaire.

**Results:**

In 2017–2018, 235 prisoners died by suicide. The suicide rate was 16.8/10 000 person-years. Among suicide cases, 94.9% were male, 27.2% were under 30, 25.1% were aged 30 to 39, 27.7% were aged 40 to 49 and 20.0% were 50 or older. At the time of suicide, 48.5% were on custodial remand. Incarceration is associated with a threefold increase in the frequency of anxio-depressive disorders (24.6% in prison versus 8.2% before prison). The week before the suicidal act, 60% of prisoners visited the health unit and a significant event was detected for 61% of all cases. Suicide was less than 1 week after prison entry for 11.9% of prisoners, corresponding to a suicide rate 6.4 (CI_95%_ [4.3 – 9.5]) times higher than for the remaining time in prison, and was more than 1 year after entry for 33.7% of them.

**Conclusions:**

The high frequency of events the week before suicide in our study suggests that events in prison could play a role in the occurrence of suicides. Comparative studies are needed to further explore the time association between events and suicide in prison. As most of prisoners who died by suicide visited the health unit the week before suicide, the identification of triggering factors could help psychiatrists and other health professionals to assess the short-term risk of suicide and to implement preventive measures.

**Supplementary Information:**

The online version contains supplementary material available at 10.1186/s12888-021-03653-w.

## Background

### International background

Suicide is a leading cause of mortality in prisons worldwide [1, 2] and suicide rates among prisoners are at least three times higher for men and 9 times higher for women than in the general population [3]. A recent meta-analysis computed the results of 77 studies assessing individual risk factors of suicide in prison, comparing prisoners who died by suicide with other prisoners [4]. The main risk factors identified were health factors such as current psychiatric diagnosis [5–11], in particular depression [7, 12], alcohol misuse [5, 8, 10], previous suicide attempt [8–11] and suicidal ideation [9, 11]. Additional factors included single-cell occupancy [5, 9, 12], remand status [5, 10, 13–25], and a charge or conviction for homicide [5, 6, 12, 19, 21, 26–32]. At the prison level, suicide was found to be associated with lower levels of purposeful activities [33] and in higher security prisons [34, 35]. In case studies, high percentages of suicides were found during the first week of imprisonment [13, 36, 37]. Additionally, specific events –related to imprisonment or family – which individuals experience could also be associated with suicide risk, but this issue has received little attention.

### Situation in France

France is characterized by a medium rate of incarceration among European countries. On the 31th of January 2020, there were 105.3 people in prison per 100 000 inhabitants, corresponding to 70 651 prisoners [38]. There were 78 742 admissions in prison during 2019, leading to an indicator of the average length of imprisonment equal to 10.8 months in France. However, the lengths of imprisonment are very heterogeneous and 57% of imprisonments are less than 6 months [39]. France has one of the highest prison suicide rates among high-income countries [3], estimated at 17.0 per 10 000 person-years in 2019 [38]. In recent years, two reports on suicide among prisoners have been produced at the request of the government in order to assess the efficacy of existing preventive measures and propose new ones [40, 41]. The authors accounted for the available evidence and made respectively 17 and 20 recommendations, including training of prison staff on suicide risk assessment, reduction access to suicide methods and postvention [42]. Furthermore, several national plans on suicide or health of prisoners addressed this issue [43–46]. One of their main objectives is to improve the quality of data on suicide in prisons in order to strengthen suicide prevention. This political demand led to the establishment of a public epidemiological surveillance program assessing suicide in prison, the first results of which are presented in this article.

### Aims

Few studies have presented detailed descriptive data on prisoners who died by suicide [13, 37, 47]. Studies looking for risk factors have been conducted in France [19, 25, 48] but there is little data on the circumstances of suicide [49] and health characteristics of prisoners who died by suicide have never been investigated. Yet, such data can be helpful in determining which factors to assess in comparative studies and to adapt prevention measures. The main objective of this study is to describe the sociodemographic, penal, and health characteristics of French prisoners who died by suicide as well as the circumstances of the suicidal act. A secondary objective is to compare the suicide rate during the first week of incarceration with the suicide rate during the remaining time in prison.

## Materials and methods

### Participants and settings

The French epidemiological surveillance program of suicides in prison is supported by the National Public Health Agency, in collaboration with the National Prison Service and the health units in prisons. This program aims at an exhaustive epidemiological surveillance of prison suicides and description of the characteristics of prisoners who died by suicide. It includes all suicides of prisoners occurring in metropolitan France, French overseas regions, and territories between the 1st of January 2017 and 31th of December 2021. The present study is an intermediate analysis restricted to 2017 and 2018, which were the years for which data were available at the time of analysis. Further analysis will be undertaken when complete data is available.

In France, each time a death occurs in prison, an investigation including an autopsy is conducted to determine the cause of death. The results of the investigation are reported by the prison to the National Prison Service (Ministry of Justice) and the latter is responsible for the census of suicide cases. Suicide is defined as a self-inflicted injury with intent to die and resulting in death. Non-doubtful cases are classified as suicide within days of death. Doubtful cases of suicide are discussed in a dedicated commission at the National Prison Service and are included in the study only if the commission concludes in favour of a suicide. Deaths resulting from a hunger strike are not classified as suicides. Our study included all cases whose suicidal act occurred while being prisoner and resulted in death. Prisoners whose suicidal act or resulting death occurred outside the prison (e.g., prisoners admitted to hospital) and those who were released between the suicidal act and the resulting death were included in the study.

### Data

Sociodemographic and penal data are routinely collected by the National Prison Service for all prisoners. They were provided for this study and included additional data from the National Prison Service on the circumstances of the suicidal act. For each suicide, a double-page standardized questionnaire was sent by the National Public Health Agency to physicians working in the corresponding prison to collect health data (see Additional file 1). The questionnaire was sent on the day of death by suicide or in the following days for non-doubtful cases. For uncertain cases, the questionnaire was sent after the commission's decision of the identification of a suicide. The questionnaire was completed by the medical practitioner on the basis of the medical file. Suicide rates were computed using data on the prison population from public reports of the National Prison Service [50].

Sociodemographic variables recorded were age at the time of suicide, gender, nationality, level of education, professional status before prison, marital status, and number of children.

Penal variables were criminal status when entering prison and at the time of suicide, main offence, previous incarceration, contact with relatives (visiting rooms / paper mail or telephone / none, first week of incarceration / none, incarcerated for more than a week / semi-open facility) and the detection of a high risk of suicide while in prison by the prison administration. The criminal status is “sentenced” when all known offences have been sentenced and the appeal period is over. In the case of multiple offences, only the main offence was selected. The order of priority for offences is as follows: homicide, sexual offence, and then the offence with the longer prison sentence or incurred prison sentence for remand status.

Health data related to three different periods were collected: prior to prison, during the stay in prison, and the week before suicide. Health variables describing person’s history before prison were familial history of suicide, being a survivor of physical or sexual assault, and personal history of regular consumption of alcohol. Health variables related to the period prior to prison and to the stay in prison were: regular consumption of tobacco, cannabis, opioids, other illegal drugs, mental disorders, psychiatric or psychological follow-up, admission to a psychiatric ward, opioid substitution therapy, other psychotropic drug therapy, suicide attempts and self-harm behaviour. Characteristics of the stay in prison included: medical follow-up, somatic diseases, episodes of agitated behaviour, threats of suicide, and last medical appointment before suicide. Mental and somatic disorders were classified using the International Statistical Classification of Diseases and Related Health Problems, 10th Revision [51]. Variables describing the week before the suicidal act included psychotropic drug therapy, opioid substitution therapy, compliance with psychotropic drug therapy, suicidal ideation, an episode of agitated, impulsive or aggressive behaviour and the occurrence of an event in prison regarded as significant by the physician. Events could be related to detention, to penal situation, to family situation or to health status.

Variables related to the circumstances of suicide were the date of the fatal suicidal act, the time interval since arrival, increased monitoring by the prison administration because of a suicide risk or security reasons, location, method, the hour of discovery of the body, the presence of a suicide note and the time interval from the suicidal act to the resulting death.

### Statistical methods

All quantitative variables were transformed into categorical variables. Headcounts and percentages were computed. Data from The National Prison Service are presented for all suicide cases. Health data are presented for suicide cases with a completed health questionnaire. Sociodemographic characteristics, penal characteristics and circumstances of the suicide were compared between suicides cases with a completed health questionnaire and 1) all cases 2) cases without a completed health questionnaire.

Distributions of suicides according to the month of the year, day of the week, and time of the day were tested against a homogeneous distribution. A chi-square test was used when all expected cases were greater than or equal to 5 and a fisher test was used in other cases. A difference was significant if *p* < 0.05.

Incidence suicide rates for 2017–2018 were calculated by dividing the number of suicides by person-years at risk (PY). PY during the first week of incarceration were estimated from the number of admissions in prison during the observation period. Each admission was considered to contribute 1 person-week. This estimation was based on the assumption that both the proportion of incarcerations of less than one week and the bias related to the first week of incarceration which overlap the beginning and the end of the observation period were negligible. PY for the remaining time in prison were obtained as the difference between the total number of PY and PY during the first week. The total number of PY was calculated by multiplying the average prison population by the duration of the observation period [52]. The average prison population was estimated by the average of the prison population on the 1st of each month from the 1st of January 2017 to the 1st of January 2019. 95% confidence intervals were computed for suicide rates and for the suicide rate ratio [53].

Analyses were carried out with R software (R version 3.5.1).

## Results

### Study population

**Fig. 1:**
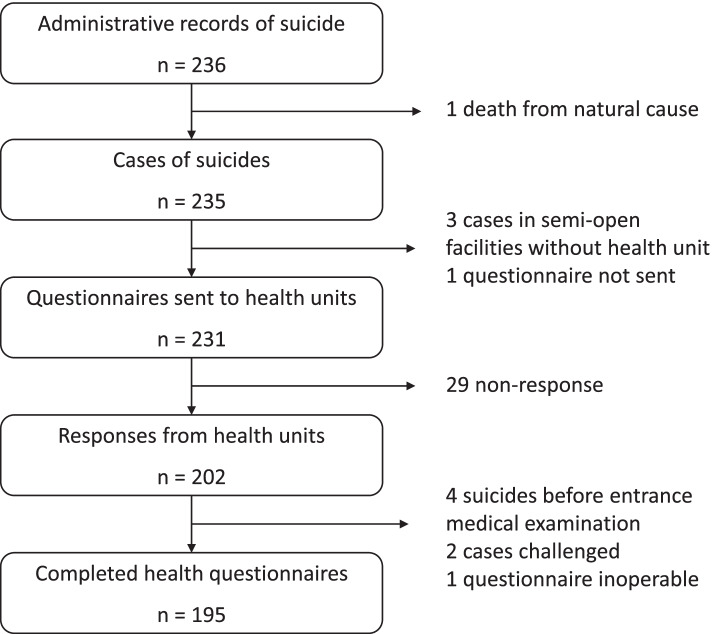
Flow chart of the study

Two hundred and thirty-six cases were registered between 2017 and 2018 (Fig. 1). One case was excluded as the death certificate mentioned a natural cause. Three cases occurred in semi-open facilities without a health unit and 1 questionnaire was not sent, resulting in 231 questionnaires sent to health units. The response rate of health units was 87%. Four suicides occurred before the entrance medical examination. Two cases were challenged by health units and were maintained in the study after consultation of all available information by the authors. Health variables were informed for 195 cases.

### Sociodemographic, penal, and custodial characteristics

Sociodemographic and penal characteristics of suicide cases are presented in Table 1. Of the 235 suicides which occurred in 2017–2018, 94.9% were male, 27.2% were under 30, 25.1% were aged 30 to 39, 27.7% were aged 40 to 49 and 20.0% were 50 or older. More than half were unemployed before imprisonment (51.5%), 18.3% were foreigners, 40.4% lived with a partner, and 57.9% had children. Almost all suicide cases were on custodial remand (91.9%) when entering prison and half were still in that situation (48.5%) at the time of suicide. The main offence was in most cases homicide (22.6%), sexual offences (22.6%), assault (19.6%), or burglary/theft (17.4%) and half of the suicide cases had previously been incarcerated (51.1%). In prison, 39.1% had contact with relatives in the visiting rooms, 13.2% had contacts only by paper mail or telephone, and 43.0% did not have any contact, but more than a quarter of the latter had just entered prison. A high risk of suicide had been detected for 25.5% of prisoners.

**Table 1:** Sociodemographic and penal characteristics of suicide cases (*n* = 235)

	n	%
Male gender	223	94.9
Age at the time of suicide (years)		
< *18*	1	0.4
* 18–29*	63	26.8
* 30–39*	59	25.1
* 40–49*	65	27.7
* 50–59*	33	14.0
≥ *60*	14	6.0
French nationality	192	81.7
Education		
* None or primary school*	10	4.3
* College*	92	39.1
* High school*	66	28.1
* University studies*	18	7.7
* Missing*	49	20.8
Employment before prison		
* Employed*	93	39.6
* Unemployed*	121	51.5
* Retired*	3	1.3
* Missing*	18	7.6
Marital status		
* Single*	109	46.4
* Lives with a partner (including married)*	95	40.4
* Divorced*	24	10.2
* Widow(er)*	5	2.1
* Missing*	2	0.9
Children		
* 0*	88	37.4
≥ *1*	136	57.9
* Missing*	11	4.7
Remand status when entering prison	216	91.9
Remand status at the time of suicide	114	48.5
Main offence		
* Homicide*	53	22.6
* Sexual offence*	53	22.6
* Assault*	46	19.6
* Burglary or theft offence*	41	17.4
* Drug-related offence*	16	6.8
* Other*	26	11.1
First incarceration	115	48.9
Contact with relatives in prison		
* Visiting rooms*	92	39.1
* Paper mail and/or telephone*	31	13.2
* None, first week of incarceration*	30	12.8
* None, incarcerated for more than a week*	71	30.2
* Semi-open facility*	4	1.7
* Missing*	7	3.0
Detection of a high risk of suicide while in prison		
* Yes*	60	25.5
* No*	137	58.3
* Missing*	38	16.2

### Health characteristics

Health characteristics are presented in Table 2 for the 195 suicides with a completed health questionnaire. No significant difference was found between cases with health data and all suicides cases. However, cases without health data were less likely to be in touch with their relatives (*p* = 0.003) and were more likely to die by self-poisoning/overdose (*p* = 0.044) than cases with health data (see Additional file 2).

**Table 2:** Health characteristics of suicide cases with a completed heath questionnaire (*n* = 195)

	History before prison		During the stay in prison		The week before suicide	
	n	%	n	%	n	%
**Traumatic life events**						
Family history of suicide						
* Yes*	14	7.2	-	-	-	-
* No*	68	34.9	-	-	-	-
* Missing*	113	57.9	-	-	-	-
Physical abuse						
* Yes*	26	13.3	-	-	-	-
* No*	62	31.8	-	-	-	-
* Missing*	107	54.9	-	-	-	-
Sexual abuse						
* Yes*	13	6.7	-	-	-	-
* No*	70	35.9	-	-	-	-
* Missing*	112	57.4	-	-	-	-
**Substance use**						
Regular smoking						
* Yes*	144	73.8	127	65.1	-	-
* No*	39	20.0	36	18.5	-	-
* Missing*	12	6.2	32	16.4	-	-
Regular alcohol consumption						
* Yes*	74	37.9	-	-	-	-
* No*	98	50.3	-	-	-	-
* Missing*	23	11.8	-	-	-	-
Regular cannabis consumption						
* Yes*	74	37.9	27	13.8	-	-
* No*	97	49.7	88	45.1	-	-
* Missing*	24	12.3	80	41.0	-	-
Regular opioid consumption						
* Yes*	29	14.9	4	2.1	-	-
* No*	142	72.8	111	56.9	-	-
* Missing*	24	12.3	80	41.0	-	-
Regular consumption of other drugs						
* Yes*	43	22.1	6	3.1	-	-
* No*	121	62.1	106	54.3	-	-
* Missing*	31	15.8	83	42.6	-	-
**Diseases**						
Somatic disorders^a^						
* Any disorder*	-	-	90	46.2	-	-
* Disease of the circulatory system*	-	-	16	8.2	-	-
* Disease of the musculoskeletal system*	-	-	25	12.8	-	-
* No disorder*	-	-	93	47.7		
* Missing*	-	-	12	6.2	-	-
Mental disorders^a^						
* Any disorder*	80	41.0	110	56.4	-	-
* Psychotic disorder*	16	8.2	22	11.3	-	-
* Bipolar disorder*	7	3.6	7	3.6	-	-
* Anxio-depressive disorder*	16	8.2	48	24.6	-	-
* Personality disorder*	16	8.2	28	14.4	-	-
* No disorder*	97	49.7	71	36.4	-	-
* Missing*	18	9.2	14	7.2	-	-
**Health care**						
Regular follow-up by the health unit						
* Yes*	-	-	135	69.2	-	-
* No*	-	-	55	28.2	-	-
* Missing*	-	-	5	2.6	-	-
Psychological/psychiatric follow-up						
* Yes*	111	56.9	112	57.4	-	-
* No*	61	31.3	68	34.9	-	-
* Missing*	23	11.8	15	7.7	-	-
Admission to a psychiatric ward						
* Yes*	66	33.8	56	28.7	-	-
* No*	105	53.8	128	65.6	-	-
* Missing*	24	12.4	11	5.6	-	-
**Psychotropic treatments**						
Opioid substitution treatment^b^						
* Yes*	25	12.8	17	8.7	14	7.2
* No*	143	73.3	163	83.6	171	87.7
* Missing*	27	13.9	15	7.7	10	5.1
Other psychotropic treatment^b^						
* Yes*	96	49.2	106	54.4	114	58.5
* No*	73	37.5	76	39.0	69	35.4
* Missing*	26	13.3	13	6.7	12	6.1
Observance to psychotropic treatments (*n* = 115)						
* Yes*	-	-	-	-	76	66.1
* No*	-	-	-	-	5	4.3
* Missing*	-	-	-	-	34	29.6
**Self-harm and suicidal behaviour**						
Self-harm behaviour						
* Yes*	28	14.4	27	13.9	-	-
* No*	120	61.5	149	76.4	-	-
* Missing*	47	24.1	19	9.7	-	-
Suicide attempt						
* Yes*	58	29.7	43	22.1	-	-
* No*	104	53.3	142	72.8	-	-
* Missing*	33	16.9	10	5.1	-	-
Suicide threat						
* Yes*	-	-	48	24.6	-	-
* No*	-	-	130	66.7	-	-
* Missing*	-	-	17	8.7	-	-
Suicidal ideation						
* Yes*	-	-	-	-	34	17.4
* No*	-	-	-	-	139	71.3
* Missing*	-	-	-	-	22	11.4
**Others**						
Episode of agitated behaviour^c^						
* Yes*	-	-	37	19.0	35	17.9
* No*	-	-	143	73.3	138	77.8
* Missing*	-	-	15	7.7	22	11.3
Time interval between last visit to health unit and suicide						
< *1 week*	-	-	117	60.0	-	-
* From 1 week to 1 month*	-	-	46	23.6	-	-
> *1 month*	-	-	25	12.8	-	-
* Missing*	-	-	7	3.6	-	-
Occurrence of a significant event						
* Yes, a custodial event*	-	-	-	-	58	29.7
* Yes, a penal event*	-	-	-	-	34	17.4
* Yes, a family event*	-	-	-	-	19	9.7
* Yes, a health event*	-	-	-	-	1	0.5
* Yes, of unknown origin*	-	-	-	-	7	3.6
* No event detected*	-	-	-	-	76	39

^a^The same person may have several disorders. ^b^In prison: treatment longer than a week. ^c^The week before suicide: episode of agitated, impulsive or aggressive behaviour

A family history of suicide was reported for 14 cases (7.2%), a history of physical abuse for 26 (13.3%), and a history of sexual abuse for 13 (6.7%). Except for tobacco, substance use was reported less often in prison than before prison. Respectively, 74 (37.9%), 29 (14.9%), and 43 (22.1%) of study participants had a history of regular consumption of cannabis, opioids, and other illegal drugs before prison, versus respectively 27 (13.8%), 4 (2.1%) and 6 (3.1%) in prison. While in prison, the use of at least one psychoactive substance was reported by 129 (66.2%) persons who died by suicide.

A history of psychiatric disorders was reported for 80 (41.0%) persons who died by suicide, whereas 110 (56.4%) had psychiatric problems while in prison. The number of persons with anxiety and depression increased from 16 (8.2%) prior to prison to 48 (24.6%) in prison. While in prison, the use of at least one substance was reported by 79 (71.9%) persons who died by suicide and had a psychiatric disorder and 28 (58.3%) persons who died by suicide and had an anxio-depressive disorder. Furthermore, the most frequent somatic disorders diagnosed in prison were disease of the musculoskeletal system (12.8%) and disease of the circulatory system (8.2%).

The number of suicide cases with psychiatric or psychological follow-up was equivalent before (*n* = 111, 56.9%) and in prison (*n* = 112, 57.4%). Admission to psychiatric ward was reported for 66 (33.8%) prisoners before prison and 56 (28.7%) in prison. Opioid substitution treatment and other psychotropic treatments were, respectively, reported for 12.8% and 49.2% of cases before prison, 8.7% and 54.4% in prison, and 7.2% and 58.5% the week before suicide.

Self-harm behaviour and suicide attempts were reported in almost as many cases during the time interval from entry in prison to suicide (respectively, 13.9% and 22.1%) as during the whole life before prison (respectively 14.4% and 29.7%). Suicide threat was reported for 48 (24.6%) cases in prison. The week before prison, suicidal ideation was reported for 34 (17.4%) and an episode of agitated, impulsive or aggressive behaviour was reported for 35 (17.9%) cases.

More than half of the cases visited the health unit the week before suicide (*n* = 117, 60.0%). Moreover, a significant event has been detected the week before suicide for 119 (61.0%) cases. This event was most often a custodial event (*n* = 58, 27.9%) such as entry in prison, transfer between two facilities or entry in the punishment block, a penal event (*n* = 34, 17.4%) such as conviction judgement or refusal of adapting a prison sentence or a family event (*n* = 19, 9.7%) such as divorce.

### Circumstances of the suicidal act

The circumstances of the suicidal act are presented in Table 3. Of the 235 suicides, the suicidal act happened during the first week of incarceration for 11.9% of prisoners, between 1 week and 1 month after entry for 8.5%, between 1 and 6 months for 31.5%, between 6 and 12 months for 14.5% and after 1 year for 33.7%. More than half of cases were subject to increased monitoring.

**Table 3:** Circumstances of the suicidal act (*n* = 235)

	n	%
Time interval from arrival to the suicidal act		
< *1 week*	28	11.9
* 1 week to 1 month*	20	8.5
* 1 to 6 months*	74	31.5
* 6 to 12 months*	34	14.5
* 1 to 5 years*	61	26
≥ *5 years*	18	7.7
Increased monitoring		
* Yes*	127	54.0
* No*	93	39.6
* Missing*	15	6.4
Location		
* Common single cell*	81	34.5
* Common shared cell*	55	23.4
* Arrival section*	40	17
* Punishment block*	32	13.6
* Non-disciplinary solitary confinement*	6	2.6
* Psychiatric health unit in prison*	7	3
* Hospital (outside prison)*	13	5.5
* Private home (permission)*	1	0.4
Method		
* Hanging/Self-strangulation*	214	91.1
* Self-poisoning/Overdose*	9	3.8
* Suffocation*^*a*^	6	2.6
* Cutting*	5	2.1
* Fire*	1	0.4
Time of discovery		
* 3 a.m.—9 a.m*	67	28.5
* 9 a.m.—3 p.m*	53	22.6
* 3 p.m.—9 p.m*	73	31.1
* 9 p.m.—3 a.m*	41	17.4
* Missing*	1	0.4
Suicide note		
* Yes*	96	40.9
* No*	118	50.2
* Missing*	21	8.9
Time interval from suicidal act to death		
* 0 day*	194	82.6
* 1 to 3 days*	24	10.2
* 4 to 22 days*	17	7.2

^a^Use of a plastic bag around the head

The number of suicides did not vary significantly according to the month of the year (*p* = 0.685). However, they were found to be more frequent on Monday or Tuesday (average 18.3%) than for the rest of the week (average 12.7%, *p* = 0.006). Additionally, after the suicidal act, the time of discovery varied significantly along the day. Notably, prisoners were more often discovered between 3 p.m. and 9 p.m. (31.1%) and less often between 9 p.m. and 3 a.m. (17.4%, *p* = 0.014).

More than 40% of the suicidal acts occurred outside common cells (*n* = 99, 42.1%). Forty (17%) suicidal acts occurred in the arrival section, 32 (13.6%) in the punishment block, and 20 (8.5%) in health units inside (*n* = 7) or outside (*n* = 13) prisons. Among the cases in common shared cells, 39 (71%) waited for their cellmate to leave the cell before acting. Date of entry was known for 30 out of 32 cases in the punishment block and among them 11 suicides were the day of entry. The method was hanging or self-strangulation for almost all cases (*n* = 214, 91.1%). Bed linen was used as a ligature for 140 (65.4%) of them (see Additional file 3). A suicide note was found for 96 (40.9%) cases and 41 (17.4%) did not die the day of the suicidal act but in the following days.

### Suicide rates

The overall suicide rate in prison was 16.8 per 10 000 PY (CI_95%_ [14.7 – 19.0], Table 4). Suicide rates were 96.7 per 10 000 PY (CI_95%_ [63.7 – 136.7]) in the first week of incarceration and 15.1 per 10 000 PY (CI_95%_ [13.1 – 17.2]) for the remaining time in prison. Suicide rate was 6.4 (CI_95%_ [4.3 – 9.5]) times higher during the first week of incarceration than for the remaining time in prison. Suicides rates according to some characteristics of the cases are presented in an additional file (see Additional file 4).

**Table 4:** Suicide incidence rate according to the stage of incarceration

Stage of incarceration	Suicides	PY	Suicide rate /10 000 PY	95% confidence interval
Whole incarceration	235	139 635.6	16.8	[14.7; 19.0]
During the 1st week of incarceration	28	2 894.5	96.7	[63.7; 136.7]
After the 1st week of incarceration	207	136 741.1	15.1	[13.1; 17.2]

## Discussion

### Main results

In 2017–2018, 235 prisoners died by suicide in France. The suicide rate was 16.8/10 000 PY. Incarceration is associated with a threefold increase in anxio-depressive disorders (24.6% versus 8.2% before prison). The week before suicide, 60% of prisoners visited the health unit and a significant event was detected in 61% of all cases. The suicidal act was less than 1 week after entry for 11.9% of prisoners, corresponding to a suicide rate 6.4 times higher than for the remaining time in prison, and was more than 1 year after entry for 33.7% of them.

### Anxio-depressive disorder in prison

Anxio-depressive disorders were reported three times more frequently in prison than before prison. It is unlikely that this increase reflects the diagnosis in prison of preexisting anxio-depressive disorders, as other mental disorders remained stable or increased much less in prison compared to prior prison. Furthermore, anxio-depressive disorders are independent risk factors of suicide and several criteria converge in favor of a causal relationship [54]. Current psychiatric diagnosis (OR = 6.4) and depression (OR = 4.9) are consistently associated with suicide in prison [4–11, 55]. The risk of suicide increases with the severity of depression [56], depression precedes suicide, and the relationship is plausible on the pathophysiological level. Thus, our results suggest that imprisonment is associated with an increased risk of suicide. This interpretation is consolidated by the fact that self-harm behaviour and suicide attempts were reported in almost as many cases during the time interval from entry in prison to suicide as during the whole life span before prison. However, this additional risk of suicide cannot be attributed to prison alone. Imprisonment is often the culmination of a process which may involve, sometimes in a very condensed manner, the commission of the offence, arrest, police custody, court proceedings, and imprisonment. This process leads to stigmatization, loss of employment and a family rejection or guilt can be added in case of a violent crime, especially when the victim is a member of the family. These are all potentially traumatic events which may help to explain the increased risk of suicide in prison. Additional studies dedicated to the comparison of mental health between the period before prison and the period in prison among prisoners are needed to further explore this issue.

### Significant events the week before suicide

A significant event was detected the week before suicide in more than half of the cases and three-quarters of them were custodial or penal events. The proportion of penal events is slightly lower than what was found in Belgium (21.1%) in the 5 days before or after suicide among 262 cases [13]. Additionally, entry in the punishment block appears to be a recurrent event preceding suicide. In France, the solitary confinement in the punishment block can last between 1 and 30 days. In our study, 11 suicides occurred the day of entry in the punishment block and 32 suicides (13.6%) occurred in that place. Moreover, the suicide risk in the punishment block was found to be 15.7 times higher than in common cells in another study [19]. Entry in the punishment block is a significant event not only because of isolation but also because of the associated consequences. This entry is often preceded by a conflict and is associated with other forthcoming measures, such as removal of sentence reduction credits, an additional sentence, refusal to adapt sentence, loss of prison work, refusal of permission or transfer to another prison.

More generally, the high frequency of significant events before suicide invites to wonder about the role played by potential triggering factors in the occurrence of suicides in prison. To our knowledge, the association between events and suicide in prison has never been assessed. Our results are restricted to suicide cases and it cannot be excluded that non-suicide prisoners experienced these events to a similar frequency. Thus, longitudinal comparative studies are needed to further explore the time association between events during imprisonment and suicide. The exploration of potential triggering factors may help in targeting suicide prevention measures focused on the suicidal act. Moreover, in the case of causal arguments for a modifiable trigger factor, prevention measures could target the event itself.

### Timing of suicide

In our study, the suicidal act occurred during the first week of incarceration for 11.9% of cases. High percentages were found as well in other studies. In Belgium, between 2000 and 2016, 15.6% occurred the first week [13]. In England and Wales, 26% out of 766 suicides in prison that occurred between 1999 and 2007 were in the first week of incarceration [36]. In Australia, 17.9% of self-inflicted deaths in prison between 1999 and 2013 (*n* = 240) occurred in the first week [37]. However, these percentages cannot be accurately compared because the average length of imprisonment differs across countries: the lower is the average length, the higher is the expected percentage of suicides during the first week under the assumption of a homogeneous distribution of suicides across time. To be comparable, suicides of the first week need to be standardized and one way of doing that is to compute suicide incidence rates. In our study, the suicide rate was 96.7/10 000 PY in the first week and was 6.4 times higher than for the remaining time in prison.

In the same way, the risk of late suicide does not seem high at first glance, as only 33.7% of them occurred more than 1 year after entry. However, only 23% of all imprisonments in France last more than 1 year [39]. Thus, a minority of suicides occurred more than 1 year after entry mainly because few prisoners are still in prison, and we do not know if the individual risk is higher or lower than before 1 year. Additional research that accounts for PY at each stage of incarceration are needed.

After the suicidal act, the prisoners were more often discovered between 3 p.m. and 9 p.m. (31.1%) than at other times of the day. When dividing the day into the same 6-h time slots, suicides were also more frequent between 3 p.m. and 9 p.m. in Belgium (32.2%) [13]. In the United-States of America, the same time slot was found for 37.4% of suicides in a study [57] but only for 21.5% in another study [58].

### Strengths and limits

One strength of our study is to gather data on more than 200 suicides in prison, giving enough power to bring significant results on the timing of suicide. Moreover, our study is the first study on suicide in prisons exhaustive at the national level in France. The exhaustive nature of the study limits the selection bias. Another strength of our study is to provide detailed data on the health characteristics of prisoners who died by suicide and on the circumstances of the suicidal act.

The main limit of our study lies in the quality of the data. Our data are based on administrative data and on the medical file. These are routine data that were not collected for research purpose and some of them may lack of reliability. In particular, some health characteristics present many missing data. In France, the entrance medical examination is compulsory and carried out for all prisoners the day of entry (except for weekends) and this limits missing data. Still, some health characteristics are likely to be underreported because of prisoners' reluctance to disclose them or because of undetected mental disorders. However, this problem of data quality is common among studies on suicide as the deceased cannot be interviewed. This issue is also difficult to anticipate as suicides are statistically scarce. One way to get around this issue is to interview survivors of near-lethal suicide attempts [13, 59–63]. They may be a good proxy for death by suicide, as the sociodemographic and penal characteristics of prisoners who made near-lethal suicide attempts were found to be similar to those who died by suicide [64]. Another limitation of the study is that the commission of the National Prison Service has not discussed doubtful cases of 2018 yet. Thus, the assessment of cases may be incomplete. However, only two doubtful cases of 2017 and five doubtful cases of 2016 were classified as suicide, which leads us to think that at most few cases are missing in 2018 and that this issue has little impact on our results.

## Conclusion

The high rate of suicide during the first week of detention argues for the reinforcement of preventive measures upon entry into prison. In addition, the high frequency of events the week before suicide in our study suggests that events in prison could play a role in the occurrence of suicides. Comparative studies are needed to further explore the time association between events and suicide in prison. As most of prisoners who died by suicide visited the health unit the week before suicide, the identification of triggering factors could help psychiatrists and other health professionals to assess the short-term risk of suicide and to implement preventive measures.

## Supplementary Information

**Additional file 1:** Health questionnaire of the study in English and in French.

**Additional file 2:** **Table 1** Sociodemographic, penal characteristics and circumstances of suicide of cases according to the collection of health data.

**Additional file 3:** **Tables 2 and 3** Ligature points and ligature for cases deceased by hanging/self-strangulation.

**Additional file 4:** **Table 4** Suicide incidence rates according to some characteristics of the prisoners.

## Data Availability

The data are not publicly available. The agreement signed between the National Public Health Agency and the National Prison Service stipulates that the data collected for this specific study cannot be used for other purposes due to the sensitivity of individual data on prisoners.

## Abbreviation

PY: Person-Years at risk

## References

[1] Konrad N, Daigle MS, Daniel AE, Dear GE, Frottier P, Hayes LM (2007). Preventing suicide in prisons, part I. Recommendations from the International Association for Suicide Prevention Task Force on Suicide in Prisons. Crisis.

[2] Rabe K (2012). Prison structure, inmate mortality and suicide risk in Europe. Int J Law Psychiatry.

[3] Fazel S, Ramesh T, Hawton K (2017). Suicide in prisons: an international study of prevalence and contributory factors. Lancet Psychiatry.

[4] Zhong S, Senior M, Yu R, Perry A, Hawton K, Shaw J (2021). Risk factors for suicide in prisons: a systematic review and meta-analysis. Lancet Public Health.

[5] Humber N, Webb R, Piper M, Appleby L, Shaw J (2013). A national case–control study of risk factors for suicide among prisoners in England and Wales [corrected]. Soc Psychiatry Psychiatr Epidemiol.

[6] Brittain J, Axelrod G, Venters H (2013). Deaths in New York City jails, 2001–2009. Am J Public Health.

[7] Baillargeon J, Penn JV, Thomas CR, Temple JR, Baillargeon G, Murray OJ (2009). Psychiatric disorders and suicide in the nation’s largest state prison system. J Am Acad Psychiatry Law.

[8] Blaauw E, Kerkhof AJFM, Hayes LM (2005). Demographic, Criminal, and Psychiatric Factors Related to Inmate Suicide. Suicide Life Threat Behav.

[9] Fruehwald S, Matschnig T, Koenig F, Bauer P, Frottier P (2004). Suicide in custody: Case-control study. Br J Psychiatry.

[10] Winter MM (2003). County Jail Suicides in a Midwestern State: Moving Beyond the Use of Profiles. Prison J.

[11] Dahle K-P, Lohner JC, Konrad N (2005). Suicide Prevention in Penal Institutions: Validation and Optimization of a Screening Tool for Early Identification of High-Risk Inmates in Pretrial Detention. Int J Forensic Ment Health.

[12] Lupei RA (1981). Jail Suicides: Demographic and Behavioral Factors Postdictive of the Completed Act.

[13] Favril L, Wittouck C, Audenaert K, Vander LF (2019). A 17-Year National Study of Prison Suicides in Belgium. Crisis.

[14] Bird SM (2008). Changes in male suicides in Scottish prisons: 10-year study. Br J Psychiatry.

[15] Radeloff D, Lempp T, Herrmann E, Kettner M, Bennefeld-Kersten K, Freitag CM (2015). National total survey of German adolescent suicide in prison. Eur Child Adolesc Psychiatry.

[16] Austin AE, van den Heuvel C, Byard RW (2014). Prison suicides in South Australia: 1996–2010. J Forensic Sci.

[17] O’Driscoll C, Samuels A, Zacka M (2007). Suicide in New South Wales Prisons, 1995–2005: towards a better understanding. Aust N Z J Psychiatry.

[18] Gauthier S, Reisch T, Bartsch C (2015). Swiss Prison Suicides Between 2000 and 2010. Crisis.

[19] Duthé G, Hazard A, Kensey A, Pan Ké Shon J-L (2013). Suicide among male prisoners in France: A prospective population-based study. Forensic Sci Int.

[20] Fruehwald S, Frottier P, Eher R, Gutierrez K, Ritter K (2000). Prison Suicides in Austria, 1975–1997. Suicide Life Threat Behav.

[21] Dooley E (1990). Prison suicide in England and Wales, 1972–87. Br J Psychiatry J Ment Sci.

[22] Snow L, Paton J, Oram C, Teers R (2002). Self-inflicted deaths during 2001: an analysis of trends. Br J Forensic Pract.

[23] Hurley W (1989). Suicides by prisoners. Med J Aust.

[24] Backett SA (1987). Suicide in Scottish prisons. Br J Psychiatry J Ment Sci.

[25] Bourgoin N (1993). La mortalité par suicide en prison. [Mortality due to suicide in prison]. Rev Epidemiol Sante Publique.

[26] Radeloff D, Stoeber F, Lempp T, Kettner M, Bennefeld-Kersten K (2019). Murderers or thieves at risk? Offence-related suicide rates in adolescent and adult prison populations. PloS One.

[27] DuRand CJ, Burtka GJ, Federman EJ, Haycox JA, Smith JW (1995). A quarter century of suicide in a major urban jail: implications for community psychiatry. Am J Psychiatry.

[28] Laishes J (1997). Inmate suicides in the Correctional Service of Canada. Crisis.

[29] Anno BJ (1985). Patterns of suicide in the Texas Department of Corrections 1980–1985. J Prison Jail Health.

[30] Salive ME, Smith GS, Brewer TF (1989). Suicide mortality in the Maryland state prison system, 1979 through 1987. JAMA.

[31] Kerkhof AJ, Bernasco W (1990). Suicidal behavior in jails and prisons in The Netherlands: incidence, characteristics, and prevention. Suicide Life Threat Behav.

[32] Bogue J, Power K (1995). Suicide in Scottish Prisons, 1976–93. J Forensic Psychiatry.

[33] Leese M, Thomas S, Snow L (2006). An ecological study of factors associated with rates of self-inflicted death in prisons in England and Wales. Int J Law Psychiatry.

[34] Dye MH (2010). Deprivation, importation, and prison suicide: Combined effects of institutional conditions and inmate composition. J Crim Justice.

[35] van Ginneken EFJC, Sutherland A, Molleman T (2017). An ecological analysis of prison overcrowding and suicide rates in England and Wales, 2000–2014. Int J Law Psychiatry.

[36] Humber N, Piper M, Appleby L, Shaw J (2011). Characteristics of and trends in subgroups of prisoner suicides in England and Wales. Psychol Med.

[37] Willis M, Baker A, Cussen T, Patterson E. Self-inflicted deaths in Australian prisons. Trends Issues Crime Crim Justice [Internet]. 2016;513. Available from: https://aic.gov.au/publications/tandi/tandi513

[38] Aebi M, Tiago M. SPACE I -2020 [Internet]. Council of Europe Annual Penal Statistics: Prison populations.; 2020 p. 134. Available from: https://wp.unil.ch/space/files/2021/04/210330_FinalReport_SPACE_I_2020.pdf

[39] De Bruyn F, Kensey A. Durées de détention plus longues, personnes détenues en plus grand nombre (2007–2013). Cah D’études Pénit Criminol [Internet]. 2014;(40). Available from: http://www.justice.gouv.fr/art_pix/cahiers_etudes_40_opt.pdf

[40] Terra JL. Prévention du suicide des personnes détenues. Evaluation des actions mises en place et propositions pour développer un programme complet de prévention. [Internet]. Paris : Rapport de mission à la demande du garde des Sceaux, ministre de la Justice et du ministre de la Santé, de la Famille et des Personnes Handicapées; 2003 p. 235. Available from: http://www.justice.gouv.fr/art_pix/Rappor_Terra.pdf

[41] Albrand L. La prévention du suicide en milieu carcéral [Internet]. Rapport au garde des Sceaux; 2009 p. 310. Available from: https://www.vie-publique.fr/rapport/30636-la-prevention-du-suicide-en-milieu-carceral-commission-presidee-par-le

[42] Andriessen K (2009). Can postvention be prevention?. Crisis.

[43] Ministère de la Santé et des Sports, Ministère de la Justice et des Libertés. Plan d’actions stratégiques 2010 - 2014. Politique de santé pour les personnes placées sous main de justice [Internet]. Available from: https://solidarites-sante.gouv.fr/IMG/pdf/plan_strategique_2010_2013_prise_en_charge_personnes_placees_sous_main_de_justice.pdf

[44] Ministère de la Justice et des Libertés, Ministère du Travail, de l'Emploi et de la Santé, Ministère de l'Education nationale, de la Jeunesse et de la Vie associative, Ministère de l'Agriculture, de l'Alimentation et de la Pêche, de la Ruralité et de l'Aménagement du territoire, Ministère de l'Enseignement supérieur et de la Recherche, Ministère de la Solidarité et de la cohésion sociale. Programme national d’actions contre le suicide (2011–2014) [Internet]. Available from: https://solidarites-sante.gouv.fr/IMG/pdf/Programme_national_d_actions_contre_le_suicide_2011-2014-2.pdf

[45] Ministère de la Justice, Ministère des Solidarités et de la Santé. Feuille de Route Santé des personnes placées sous main de justice 2019–2022 [Internet]. Available from: https://solidarites-sante.gouv.fr/IMG/pdf/fdr_sante__ppsmj_19_22.pdf

[46] Ministère des Affaires sociales et de la Santé, Ministère de la Justice (2017). Stratégie santé des personnes placées sous main de justice.

[47] Shaw J, Baker D, Hunt IM, Moloney A, Appleby L (2004). Suicide by prisoners: National clinical survey. Br J Psychiatry.

[48] Duthé G, Hazard A, Kensey A. Suicide in prison : a comparison between France and its European neighbours. Popul Soc [Internet]. 2009;(462). Available from: https://www.ined.fr/en/publications/editions/population-and-societies/suicide-in-prison-a-comparison-between-france-and-its-european-neighbours-en/

[49] Hazard A. Baisse des suicides en prison depuis 2002. Cah Etudes Penit Crim [Internet]. 2008;(22). Available from: http://www.justice.gouv.fr/art_pix/CahEtudesPenitCrim22.pdf

[50] Ministère de la Justice. Direction de l'administration pénitentiaire. DAP. Les chiffres clés de l’administration pénitentiaire [Internet]. [cited 2020 Dec 9]. Available from: http://www.justice.gouv.fr/prison-et-reinsertion-10036/les-chiffres-clefs-10041/

[51] World Health Organization (2017). ICD-10 International statistical classification of diseases and related health problems: tenth revision.

[52] Rothman K, Greenland S, Lash T. Modern Epidemiology 3rd edition. Lippincott Williams&Wilkins. 2008. 758 p.

[53] KJ Rothman 2012 Epidemiology: An Introduction. Oxford University Press 281 p.

[54] Hill AB (1965). The Environment and Disease: Association or Causation?. Proc R Soc Med.

[55] Ayhan G, Arnal R, Basurko C, About V, Pastre A, Pinganaud E (2017). Suicide risk among prisoners in French Guiana: prevalence and predictive factors. BMC Psychiatry.

[56] Hawton K, Casañas I Comabella C, Haw C, Saunders K (2013). Risk factors for suicide in individuals with depression: a systematic review. J Affect Disord.

[57] Boren EA, Folk JB, Loya JM, Tangney JP, Barboza SE, Wilson JS (2018). The Suicidal Inmate: A Comparison of Inmates Who Attempt Versus Complete Suicide. Suicide Life Threat Behav.

[58] Way BB, Miraglia R, Sawyer DA, Beer R, Eddy J (2005). Factors related to suicide in New York state prisons. Int J Law Psychiatry.

[59] Marzano L, Rivlin A, Fazel S, Hawton K (2009). Interviewing survivors of near-lethal self-harm: a novel approach for investigating suicide amongst prisoners. J Forensic Leg Med.

[60] Marzano L, Fazel S, Rivlin A, Hawton K (2010). Psychiatric disorders in women prisoners who have engaged in near-lethal self-harm: case-control study. Br J Psychiatry.

[61] Rivlin A, Hawton K, Marzano L, Fazel S (2010). Psychiatric disorders in male prisoners who made near-lethal suicide attempts: case-control study. Br J Psychiatry.

[62] Rivlin A, Hawton K, Marzano L, Fazel S (2013). Psychosocial characteristics and social networks of suicidal prisoners: towards a model of suicidal behaviour in detention. PLoS One.

[63] Sánchez FC, Fearn N, Vaughn MG (2018). Risk Factors Associated With Near-Lethal Suicide Attempts During Incarceration Among Men in the Spanish Prison System. Int J Offender Ther Comp Criminol.

[64] Rivlin A, Fazel S, Marzano L, Hawton K (2012). Studying survivors of near-lethal suicide attempts as a proxy for completed suicide in prisons. Forensic Sci Int.

