# Supplementary material for: Characteristics of persons who died by suicide in prison in France: 2017–2018
Source: BMC Psychiatry. 2022 Jan 4;22:11. doi: 10.1186/s12888-021-03653-w (PMC8729083; doi:10.1186/s12888-021-03653-w)
Supplement: Supplementary file 1 — Additional file 1: Health questionnaire of the study in English and in French. [file 12888_2021_3653_MOESM1_ESM.docx]

Additional file1

Table of contents

[**Article information** 2](#_Toc76980055)

[**Health questionnaire (translated in English from French)** 3](#_Toc76980056)

[**Health questionnaire (French version)** 5](#_Toc76980057)

# **Article information**

Article title

Characteristics of persons who died by suicide in prison in France: 2017-2018

Journal name

BMC Psychiatry

Authors

Alexis Vanhaesebrouck^1,2,3*^, Amélie Tostivint^4^, Thomas Lefèvre^1,2^, Maria Melchior^3^, Imane Khiredine-Medouni^5^, Christine Chan Chee^5^

1 Interdisciplinary Research Institute on Social issues (IRIS), UMR 8156-997, UFR SMBH, Université Sorbonne Paris Nord, Paris, France.

2 Department of legal and social Medicine, Hôpital Jean-Verdier (AP-HP), 93140 Bondy, France;

3 Sorbonne Université, INSERM, Pierre Louis Institute of Epidemiology and Public Health, Department of Social Epidemiology, Paris, France.

4 Ministry of Justice, National Prison Service, Health division, Paris ,France

5 National Agency of Public Health (Santé Publique France), Saint-Maurice, France

* alexis.vanhaesebrouck@iplesp.upmc.fr

# **Health questionnaire (translated from French)**

| **General indicators** | | | | | |
| --- | --- | --- | --- | --- | --- |
| Gender | 🞎 Male | 🞎 Female | | Number \|__\|__\|__\|__\|__\| | |
| Year of birth | \|__\|__\|__\|__\| | | | | |
| Date of suicidal act | \|__\|__\| / \|__\|__\| / \|__\|__\|__\|__\| | | | |  |
| Date of death (if different) | \|__\|__\| / \|__\|__\| / \|__\|__\|__\|__\| | | | |  |
| Method  *(multiple responses allowed)* | 🞎Hanging/Self-strangulation  🞎 Cutting  🞎 Cell fire | | 🞎 Self-poisoning  🞎 Other, please specify: ……………………………  🞎 Unknown | | |

| **Medical and psychiatric indicators** | | | |
| --- | --- | --- | --- |
| **History outside prison (recorded when entering prison)** | | | |
| History of psychiatric diagnosis | 🞎 Yes | 🞎 No* | 🞎 Uninformed |
| If yes, please specify: ………………………………………………………………………………………… | | | |
| History of hospitalization in psychiatry | 🞎 Yes | 🞎 No* | 🞎 Uninformed |
| History of psychiatric or psychological follow-up | 🞎 Yes | 🞎 No* | 🞎 Uninformed |
| History of psychotropic treatment (except OST) | 🞎 Yes | 🞎 No* | 🞎 Uninformed |
| History of opioid substitution therapy (OST) | 🞎 Yes | 🞎 No* | 🞎 Uninformed |
| Personal history of suicide attempt | 🞎 Yes | 🞎 No* | 🞎 Uninformed |
| Personal history of self-harm | 🞎 Yes | 🞎 No* | 🞎 Uninformed |
| History of regular consumption (> 3 months) | | | |
| - Tobacco | 🞎 Yes | 🞎 No* | 🞎 Uninformed |
| - Alcohol (>3 glasses/ day or >20 glasses/ week) | 🞎 Yes | 🞎 No* | 🞎 Uninformed |
| - Cannabis | 🞎 Yes | 🞎 No* | 🞎 Uninformed |
| - Opioid products (heroin, diverted or unprescribed opioid medications) | 🞎 Yes | 🞎 No* | 🞎 Uninformed |
| - Other drugs (including diverted medications) | 🞎 Yes | 🞎 No* | 🞎 Uninformed |
| History of physical abuse | 🞎 Yes | 🞎 No* | 🞎 Uninformed |
| History of sexual abuse | 🞎 Yes | 🞎 No* | 🞎 Uninformed |
| Family history of suicide | 🞎 Yes | 🞎 No* | 🞎 Uninformed |

| **Factors identified during detention, since entry in prison** | | | | | | | | | |
| --- | --- | --- | --- | --- | --- | --- | --- | --- | --- |
| Regular follow-up by the health unit  (consultations, care, delivery of treatments) 🞎 Yes 🞎 No* 🞎 Uninformed | | | | | | | | | |
| If yes, 🞎 (almost) every day 🞎 1 to 3 times a week 🞎 1 to 3 times a month | | | | | | | | | |
| Somatic disorders | 🞎 Yes | 🞎 No* | | | 🞎 Uninformed | | | | |
| If yes, please specify:………………………………………………………………………………………….... | | | | | | | | | |
| ………………………………………………………………………………………..……….. | | | | | | | | | |
| Mental disorders | 🞎 Yes | 🞎 No* | | | 🞎 Uninformed | | | | |
| If yes, please specify:………………………………………………………………………………………….... | | | | | | | | | |
| ………………………………………………………………………………………..……….. | | | | | | | | | |
| Regular psychiatric or psychological follow-up | | | 🞎 Yes | | | | | 🞎 No* | 🞎 Uninformed |
| Hospitalization in psychiatry | | | 🞎 Yes | | | | | 🞎 No* | 🞎 Uninformed |
| Episode of agitation (clastic crisis, exacerbation of a delusional syndrome…) | | | 🞎 Yes | | | | | 🞎 No* | 🞎 Uninformed |
| Psychotropic treatment (except OST) delivered at least 1 week | | | 🞎 Yes | | | | | 🞎 No* | 🞎 Uninformed |
| Opioid substitution therapy (OST) delivered at least 1 week | | | 🞎 Yes | | | | | 🞎 No* | 🞎 Uninformed |
| Regular consumption while in prison | | |  | | | |  | |  |
| - Tobacco | | | 🞎 Yes | | | | 🞎 No* | | 🞎 Uninformed |
| - Cannabis | | | 🞎 Yes | | | | 🞎 No* | | 🞎 Uninformed |
| - Opioid products (heroin, diverted or unprescribed opioid medications) | | | 🞎 Yes | | | | 🞎 No* | | 🞎 Uninformed |
| - Other drugs (including diverted medications) | | | 🞎 Yes | | | | 🞎 No* | | 🞎 Uninformed |
| Suicide attempt | 🞎 Yes | 🞎 Non | | | | 🞎 Uninformed | | | |
| Number if known /___/___/  Date of last suicide attempt \|___\|___\| / \|___\|___\| / \|___\|___\|___\|___\| | | | | | | | | | |
| Self-harm | 🞎 Yes | 🞎 Non | | | | 🞎 Uninformed | | | |
| Number if known /___/___/  Date of last self-harm  \|___\|___\| / \|___\|___\| / \|___\|___\|___\|___\| | | | | | | | | | |
| Suicide threat | 🞎 Yes | 🞎 Non | | 🞎 Uninformed | | | | | |
| The time between the last appointment with the health unit and the fatal suicidal act was | | | | | | | | | |
| 🞎 Less than a week 🞎 1 week to 1 month 🞎 > 1 month 🞎 Follow-up interrupted | | | | | | | | | |

| **Factors identified the week before the fatal suicidal act** | | | |
| --- | --- | --- | --- |
| Psychotropic treatment (except OST) at the time of the suicidal act | 🞎 Yes | 🞎 No* | 🞎 Uninformed |
| OST at the time of the suicidal act | 🞎 Yes | 🞎 No* | 🞎 Uninformed |
| Observance of the psychotropic treatment (including OST) | 🞎 Yes | 🞎 No* | 🞎 Uninformed |
| Suicidal ideation | 🞎 Yes | 🞎 No* | 🞎 Uninformed |
| Episode of agitation, impulsivity or aggressivity | 🞎 Yes | 🞎 No* | 🞎 Uninformed |
| Occurrence of a traumatic event (related to detention, related to the penal situation, family, health)  *If yes, please circle the corresponding type of traumatic event* | 🞎 Yes | 🞎 No* | 🞎 Uninformed |

**All comments, specifications and remarks on plain paper are welcome.**

| **Thank you for your participation**. |
| --- |

# **Health questionnaire (French version)**

| **Indicateurs généraux** | | | | | |
| --- | --- | --- | --- | --- | --- |
| Sexe : | 🞎 Homme | 🞎 Femme | | Numéro \|__\|__\|__\|__\|__\| | |
| Année de naissance : | \|__\|__\|__\|__\| | | | | |
| Date de l’acte suicidaire : | \|__\|__\| / \|__\|__\| / \|__\|__\|__\|__\| | | | |  |
| Date du décès (si différent) : | \|__\|__\| / \|__\|__\| / \|__\|__\|__\|__\| | | | |  |
| Mode opératoire du suicide :  *(plusieurs réponses possibles)* | 🞎 Pendaison  🞎 Phlébotomie  🞎 Feu de cellule | | 🞎 Ingestion médicamenteuse volontaire  🞎 Autre, préciser : ……………………………  🞎 Ne sait pas | | |

| **Indicateurs médicaux et psychiatriques** | | | |
| --- | --- | --- | --- |
| **Antécédents en milieu libre (relevés à l’entrée en détention)** | | | |
| Antécédent de diagnostic psychiatrique : | 🞎 Oui | 🞎 Non* | 🞎 Non renseigné |
| Si oui, préciser : ………………………………………………………………………………………… | | | |
| Antécédent d’hospitalisation en psychiatrie : | 🞎 Oui | 🞎 Non* | 🞎 Non renseigné |
| Antécédent de suivi psychologique ou psychiatrique : | 🞎 Oui | 🞎 Non* | 🞎 Non renseigné |
| Antécédent de traitement psychotrope (hors TSO) : | 🞎 Oui | 🞎 Non* | 🞎 Non renseigné |
| Antécédent de traitement de substitution aux opiacés (TSO) : | 🞎 Oui | 🞎 Non* | 🞎 Non renseigné |
| Antécédent personnel de tentative de suicide : | 🞎 Oui | 🞎 Non* | 🞎 Non renseigné |
| Antécédent personnel d’automutilations : | 🞎 Oui | 🞎 Non* | 🞎 Non renseigné |
| Antécédent d’une consommation régulière (pendant > 3 mois) : | | | |
| - Tabac | 🞎 Oui | 🞎 Non* | 🞎 Non renseigné |
| - Alcool (>3 verres/ jour ou >20 verres/ semaine) | 🞎 Oui | 🞎 Non* | 🞎 Non renseigné |
| - Cannabis | 🞎 Oui | 🞎 Non* | 🞎 Non renseigné |
| - Produits opiacés (héroïne, médicaments opiacés détournés ou non prescrits) | 🞎 Oui | 🞎 Non* | 🞎 Non renseigné |
| - Autres produits stupéfiants (y compris médicaments détournés) | 🞎 Oui | 🞎 Non* | 🞎 Non renseigné |
| Antécédent de maltraitance physique : | 🞎 Oui | 🞎 Non* | 🞎 Non renseigné |
| Antécédent de violence sexuelle subie : | 🞎 Oui | 🞎 Non* | 🞎 Non renseigné |
| Antécédent familial de suicide : | 🞎 Oui | 🞎 Non* | 🞎 Non renseigné |

| **Facteurs relevés en cours de détention, depuis l’incarcération** | | | | | | | | | |
| --- | --- | --- | --- | --- | --- | --- | --- | --- | --- |
| Suivi régulier par l’unité sanitaire  (consultations, soins, délivrance de traitement) : 🞎 Oui 🞎 Non* 🞎 Non renseigné | | | | | | | | | |
| Si oui, 🞎 tous les jours ou presque 🞎 1 à 3 fois / semaine 🞎 1 à 3 fois / mois | | | | | | | | | |
| Pathologies somatiques: | 🞎 Oui | 🞎 Non* | | | 🞎 Non renseigné | | | | |
| Si oui, préciser :……………………………………………………………………………………………….... | | | | | | | | | |
| ………………………………………………………………………………………..……….. | | | | | | | | | |
| Pathologies psychiatriques : | 🞎 Oui | 🞎 Non* | | | 🞎 Non renseigné | | | | |
| Si oui, préciser :……………………………………………………………………………………………….... | | | | | | | | | |
| ………………………………………………………………………………………..……….. | | | | | | | | | |
| Suivi psychologique ou psychiatrique régulier : | | | 🞎 Oui | | | | | 🞎 Non* | 🞎 Non renseigné |
| Hospitalisation en psychiatrie : | | | 🞎 Oui | | | | | 🞎 Non* | 🞎 Non renseigné |
| Episode d’agitation (crise clastique, exacerbation d’un syndrome délirant…) | | | 🞎 Oui | | | | | 🞎 Non* | 🞎 Non renseigné |
| Traitement psychotrope hors TSO délivré pendant plus d’une semaine | | | 🞎 Oui | | | | | 🞎 Non* | 🞎 Non renseigné |
| Traitement TSO délivré pendant plus d’une semaine | | | 🞎 Oui | | | | | 🞎 Non* | 🞎 Non renseigné |
| Consommation régulière, en cours de détention : | | |  | | | |  | |  |
| - Tabac | | | 🞎 Oui | | | | 🞎 Non* | | 🞎 Non renseigné |
| - Cannabis | | | 🞎 Oui | | | | 🞎 Non* | | 🞎 Non renseigné |
| - Produits opiacés (héroïne, médicaments opiacés détournés ou non prescrits) | | | 🞎 Oui | | | | 🞎 Non* | | 🞎 Non renseigné |
| - Autres produits stupéfiants (y compris médicaments détournés) | | | 🞎 Oui | | | | 🞎 Non* | | 🞎 Non renseigné |
| Tentative de suicide : | 🞎 Oui | 🞎 Non | | | | 🞎 Non renseigné | | | |
| Nombre si connu : /___/___/  Date de la dernière TS si connue : \|___\|___\| / \|___\|___\| / \|___\|___\|___\|___\| | | | | | | | | | |
| Automutilations : | 🞎 Oui | 🞎 Non | | | | 🞎 Non renseigné | | | |
| Nombre si connu : /___/___/  Date de la dernière automutilation si connue : \|___\|___\| / \|___\|___\| / \|___\|___\|___\|___\| | | | | | | | | | |
| Menace de suicide : | 🞎 Oui | 🞎 Non | | 🞎 Non renseigné | | | | | |
| Par rapport au passage à l’acte fatal, le dernier RV à l’unité sanitaire a eu lieu il y a : | | | | | | | | | |
| 🞎 Moins d’une semaine 🞎 Entre une semaine et un mois 🞎 Plus d’un mois 🞎 Suivi interrompu | | | | | | | | | |

| **Facteurs relevés dans la semaine précédant le passage à l’acte fatal** | | | |
| --- | --- | --- | --- |
| Traitement psychotrope (hors TSO) en cours au moment  du passage à l’acte | 🞎 Oui | 🞎 Non* | 🞎 Non renseigné |
| Traitement TSO en cours au moment du passage à l’acte | 🞎 Oui | 🞎 Non* | 🞎 Non renseigné |
| Observance du traitement psychotrope ou TSO prescrit : | 🞎 Oui | 🞎 Non* | 🞎 Non renseigné |
| Idéation suicidaire récente connue : | 🞎 Oui | 🞎 Non* | 🞎 Non renseigné |
| Surveillance pour risque suicidaire : | 🞎 Oui | 🞎 Non* | 🞎 Non renseigné |
| Episode ou état d’agitation / impulsivité / agressivité : | 🞎 Oui | 🞎 Non* | 🞎 Non renseigné |
| Survenue d’un événement traumatique (lié à la détention, à la situation pénale, familiale, sanitaire)  *Si oui, entourer l’évènement traumatique* | 🞎 Oui | 🞎 Non* | 🞎 Non renseigné |

**Tous commentaires, précisions ou autres remarques sur papier libre sont les bienvenus.**

| **Merci de votre participation**. |
| --- |
