# Supplementary material for: Characteristics of persons who died by suicide in prison in France: 2017–2018
Source: BMC Psychiatry. 2022 Jan 4;22:11. doi: 10.1186/s12888-021-03653-w (PMC8729083; doi:10.1186/s12888-021-03653-w)
Supplement: Supplementary file 2 — Additional file 2: Table 1 Sociodemographic, penal characteristics and circumstances of suicide of cases according to the collection of health data. [file 12888_2021_3653_MOESM2_ESM.docx]

Additional file 2

**Supplemental Table 1** Sociodemographic, penal characteristics and circumstances of suicide of cases according to the collection of health data

|  | Cases with  health data (n=195) (1) | | Cases without health data  (n=40) (2) | | All cases  (n=235) (3) | | (1)  vs  (3) | (1)  vs  (2) |  |
| --- | --- | --- | --- | --- | --- | --- | --- | --- | --- |
|  | n | % | n | % | n | % | p value | p value |  |
| Male gender | 185 | 94.9 | 38 | 95.0 | 223 | 94.9 | 1 | 1 |  |
| Age at the time of suicide (years) |  |  |  |  |  |  | 0.999 | 0.899 |  |
| *<18* | 1 | 0.5 | 0 | 0 | 1 | 0.4 |  |  |  |
| *18-29* | 53 | 27.2 | 10 | 25.0 | 63 | 26.8 |  |  |  |
| *30-39* | 49 | 25.1 | 10 | 25.0 | 59 | 25.1 |  |  |  |
| *40-49* | 52 | 26.7 | 13 | 32.5 | 65 | 27.7 |  |  |  |
| *50-59* | 27 | 13.8 | 6 | 15.0 | 33 | 14.0 |  |  |  |
| ≥*60* | 13 | 6.7 | 1 | 2.5 | 14 | 6.0 |  |  |  |
| French nationality | 156 | 80.0 | 36 | 90.0 | 192 | 81.7 | 0.746 | 0.206 |  |
| Education |  |  |  |  |  |  | 0.937 | 0.284 |  |
| *None or primary school* | 6 | 3.1 | 4 | 10.0 | 10 | 4.3 |  |  |  |
| *College* | 78 | 40.0 | 14 | 35.0 | 92 | 39.1 |  |  |  |
| *High school* | 54 | 27.7 | 12 | 30.0 | 66 | 28.1 |  |  |  |
| *University studies* | 15 | 7.7 | 3 | 7.5 | 18 | 7.7 |  |  |  |
| *Missing* | 42 | 21.5 | 7 | 17.5 | 49 | 20.8 |  |  |  |
| Employment before prison |  |  |  |  |  |  | 0.975 | 0.760 |  |
| *Employed* | 79 | 40.5 | 14 | 35.0 | 93 | 39.6 |  |  |  |
| *Unemployed* | 99 | 50.8 | 22 | 55.0 | 121 | 51.5 |  |  |  |
| *Retired* | 3 | 1.5 | 0 | 0 | 3 | 1.3 |  |  |  |
| *Missing* | 14 | 7.2 | 4 | 10.0 | 18 | 7.6 |  |  |  |
| Marital status |  |  |  |  |  |  | 0.951 | 0.406 |  |
| *Single* | 88 | 45.1 | 21 | 52.5 | 109 | 46.4 |  |  |  |
| *Lives with a partner (including married)* | 82 | 42.1 | 13 | 32.5 | 95 | 40.4 |  |  |  |
| *Divorced* | 18 | 9.2 | 6 | 15.0 | 24 | 10.2 |  |  |  |
| *Widow(er)* | 5 | 2.6 | 0 | 0 | 5 | 2.1 |  |  |  |
| *Missing* | 2 | 1.0 | 0 | 0 | 2 | 0.9 |  |  |  |
| Children |  |  |  |  |  |  | 0.810 | 0.349 |  |
| *0* | 70 | 35.9 | 18 | 45.0 | 88 | 37.4 |  |  |  |
| *≥1* | 116 | 59.5 | 20 | 50.0 | 136 | 57.9 |  |  |  |
| *Missing* | 9 | 4.6 | 2 | 5.0 | 11 | 4.7 |  |  |  |
| Remand status when entering prison | 179 | 91.8 | 37 | 92.5 | 216 | 91.9 | 1 | 1 |  |
| Remand status at the time of suicide | 94 | 48.2 | 20 | 50.0 | 114 | 48.5 | 1 | 0.974 |  |
| Main offence |  |  |  |  |  |  | 0.975 | 0.076 |  |
| *Homicide* | 50 | 25.6 | 3 | 7.5 | 53 | 22.6 |  |  |  |
| *Sexual offence* | 40 | 20.5 | 13 | 32.5 | 53 | 22.6 |  |  |  |
| *Assault* | 36 | 18.5 | 10 | 25 | 46 | 19.6 |  |  |  |
| *Burglary or theft offence* | 32 | 16.4 | 9 | 22.5 | 41 | 17.4 |  |  |  |
| *Drug-related offence* | 14 | 7.2 | 2 | 5.0 | 16 | 6.8 |  |  |  |
| *Other* | 23 | 11.8 | 3 | 7.5 | 26 | 11.1 |  |  |  |
| First incarceration | 99 | 50.8 | 16 | 40.0 | 115 | 48.9 | 0.778 | 0.286 |  |
| Contact with relatives in prison |  |  |  |  |  |  | 0.765 | 0.003 |  |
| *Visiting rooms* | 84 | 43.1 | 8 | 20.0 | 92 | 39.1 |  |  |  |
| *Paper mail and/or telephone* | 27 | 13.8 | 4 | 10.0 | 31 | 13.2 |  |  |  |
| *None, first week of incarceration* | 23 | 11.8 | 7 | 17.5 | 30 | 12.8 |  |  |  |
| *None, incarcerated for more than a week* | 54 | 27.7 | 17 | 42.5 | 71 | 30.2 |  |  |  |
| *Semi-open facility* | 1 | 0.5 | 3 | 7.5 | 4 | 1.7 |  |  |  |
| *Missing* | 6 | 3.1 | 1 | 2.5 | 7 | 3.0 |  |  |  |
| Time interval from arrival to the suicidal act | |  |  |  |  |  | 0.997 | 0.625 |  |
| *<1 week* | 22 | 11.3 | 6 | 15.0 | 28 | 11.9 |  |  |  |
| *1 week to 1 month* | 17 | 8.7 | 3 | 7.5 | 20 | 8.5 |  |  |  |
| *1 to 6 months* | 60 | 30.8 | 14 | 35.0 | 74 | 31.5 |  |  |  |
| *6 to 12 months* | 30 | 15.4 | 4 | 10.0 | 34 | 14.5 |  |  |  |
| *1 to 5 years* | 53 | 27.2 | 8 | 20.0 | 61 | 26 |  |  |  |
| ≥ *5 years* | 13 | 6.7 | 5 | 12.5 | 18 | 7.7 |  |  |  |
| Detection of a suicidal crisis |  |  |  |  |  |  | 0.791 | 0.290 |  |
| *Yes* | 53 | 27.2 | 7 | 17.5 | 60 | 25.5 |  |  |  |
| *No* | 111 | 56.9 | 26 | 65.0 | 137 | 58.3 |  |  |  |
| *Missing* | 31 | 15.9 | 7 | 17.5 | 38 | 16.2 |  |  |  |
| Increased monitoring |  |  |  |  |  |  | 1 | 1 |  |
| *Yes* | 106 | 54.4 | 21 | 52.5 | 127 | 54.0 |  |  |  |
| *No* | 78 | 40.0 | 15 | 37.5 | 93 | 39.6 |  |  |  |
| *Missing* | 11 | 4.6 | 4 | 10.0 | 15 | 6.4 |  |  |  |
| Location |  |  |  |  |  |  | 1 | 0.581 |  |
| *Common single cell* | 68 | 34.9 | 13 | 32.5 | 81 | 34.5 |  |  |  |
| *Common shared cell* | 48 | 24.6 | 7 | 17.5 | 55 | 23.4 |  |  |  |
| *Arrival section* | 32 | 16.4 | 8 | 20.0 | 40 | 17 |  |  |  |
| *Punishment block* | 25 | 12.8 | 7 | 17.5 | 32 | 13.6 |  |  |  |
| *Non-disciplinary solitary confinement* | 5 | 2.6 | 1 | 2.5 | 6 | 2.6 |  |  |  |
| *Psychiatric health unit in prison* | 6 | 3.1 | 1 | 2.5 | 7 | 3 |  |  |  |
| *Hospital (outside prison)* | 11 | 5.6 | 2 | 5.0 | 13 | 5.5 |  |  |  |
| *Private home (permission)* | 0 | 0 | 1 | 2.5 | 1 | 0.4 |  |  |  |
| Method |  |  |  |  |  |  | 0.888 | 0.044 |  |
| *Hanging/Self-strangulation* | 180 | 92.2 | 34 | 85.0 | 214 | 91.1 |  |  |  |
| *Self-poisoning/Overdose* | 4 | 2.1 | 5 | 12.5 | 9 | 3.8 |  |  |  |
| *Suffocation* | 5 | 2.6 | 1 | 2.5 | 6 | 2.6 |  |  |  |
| *Cutting* | 5 | 2.6 | 0 | 0 | 5 | 2.1 |  |  |  |
| *Fire* | 1 | 0.5 | 0 | 0 | 1 | 0.4 |  |  |  |
| Time of discovery |  |  |  |  |  |  | 0.945 | 0.629 |  |
| *3 a.m. - 9 a.m.* | 55 | 28.2 | 12 | 30.0 | 67 | 28.5 |  |  |  |
| *9 a.m. - 3 p.m.* | 47 | 24.1 | 6 | 15.0 | 53 | 22.6 |  |  |  |
| *3 p.m. - 9 p.m.* | 60 | 30.8 | 13 | 32.5 | 73 | 31.1 |  |  |  |
| *9 p.m. - 3 a.m.* | 30 | 16.9 | 8 | 20.0 | 41 | 17.4 |  |  |  |
| *Missing* | 0 | 0 | 1 | 2.5 | 1 | 0.4 |  |  |  |
| Letter left behind |  |  |  |  |  |  | 0.876 | 0.475 |  |
| *Yes* | 84 | 43.1 | 12 | 30.0 | 96 | 40.9 |  |  |  |
| *No* | 98 | 50.3 | 20 | 50.0 | 118 | 50.2 |  |  |  |
| *Missing* | 13 | 6.6 | 8 | 20.0 | 21 | 8.9 |  |  |  |
| Time interval from suicidal act to death |  |  |  |  |  |  | 0.896 | 0.331 |  |
| *0 day* | 162 | 83.1 | 32 | 80.0 | 194 | 82.6 |  |  |  |
| *1 to 3 days* | 21 | 10.8 | 3 | 7.5 | 24 | 10.2 |  |  |  |
| *4 to 22 days* | 12 | 6.1 | 5 | 12.5 | 17 | 7.2 |  |  |  |
