# Supplementary material for: Characteristics of persons who died by suicide in prison in France: 2017–2018
Source: BMC Psychiatry. 2022 Jan 4;22:11. doi: 10.1186/s12888-021-03653-w (PMC8729083; doi:10.1186/s12888-021-03653-w)
Supplement: Supplementary file 3 — Additional file 3: Tables 2 and 3 Ligature points and ligature for cases deceased by hanging/self-strangulation. [file 12888_2021_3653_MOESM3_ESM.docx]

Additional file 3

**Table of contents**

[**Supplemental Table 2. Ligature points for cases deceased by hanging/self-strangulation (n = 214) 2**](#_Toc86747459)

[**Supplemental Table 3. Ligatures for cases deceased by hanging/self-strangulation (n = 214) 3**](#_Toc86747460)

# Supplemental Table 2. Ligature points among cases deceased by hanging/self-strangulation (n = 214)

|  | n | % |
| --- | --- | --- |
| **Window** | **76** | **35.5** |
| Bar | 46 | 21.5 |
| Frame | 3 | 1.4 |
| Opening | 1 | 0.5 |
| Handle | 1 | 0.5 |
| No details | 25 | 11.7 |
| **Cell bar** | **30** | **14.0** |
| In the punishment block | 22 | 10.3 |
| Other | 8 | 3.7 |
| **Sanitary** | **29** | **13.6** |
| Door | 10 | 4.7 |
| Partition wall | 7 | 3.3 |
| Frame | 5 | 2.3 |
| Curtain rod | 3 | 1.4 |
| Door hinge | 2 | 0.9 |
| Shower head | 1 | 0.5 |
| Metal support for the shower access | 1 | 0.5 |
| **Bed** | **24** | **11.2** |
| Frame, bunkbed | 17 | 7.9 |
| Foot | 2 | 0.9 |
| No details | 5 | 2.3 |
| **Heater or line pipe** | **16** | **7.5** |
| **Closet** | **3** | **1.4** |
| Handle | 1 | 0.5 |
| Door | 1 | 0.5 |
| Rod | 1 | 0.5 |
| **Other** | **11** | **5.1** |
| Bar – no details | 3 | 1.4 |
| Ceiling light fixture | 1 | 0.5 |
| Chamber door (hospital) | 1 | 0.5 |
| Coat rack | 1 | 0.5 |
| Desk | 1 | 0.5 |
| Screw in the wall | 2 | 0.9 |
| Television stand | 2 | 0.9 |
| **Missing** | **25** | **11.7** |
|  |  |  |

# Supplemental Table 3. Ligatures among cases deceased by hanging/self-strangulation (n = 214)

|  | **n** | **%** |
| --- | --- | --- |
| **Bed linen** | **140** | **65.4** |
| Sheet | 130 | 60.7 |
| Blanket | 4 | 1.9 |
| Piece of blanket | 3 | 1.4 |
| Mattress cover | 3 | 1.4 |
| **Belt** | **15** | **7.0** |
| **Lace or cord** | **14** | **6.5** |
| Lace | 12 | 5.6 |
| Garment cord | 1 | 0.5 |
| No detail | 1 | 0.5 |
| **Other clothes** | **15** | **7.0** |
| Scarf | 3 | 1.4 |
| Jogging | 2 | 0.9 |
| Bathrobe | 2 | 0.9 |
| Sweater | 2 | 0.9 |
| T-shirt | 2 | 0.9 |
| Tracksuit | 1 | 0.5 |
| Work coat | 1 | 0.5 |
| Headband | 1 | 0.5 |
| No detail | 1 | 0.5 |
| **Electronic equipment** | **7** | **3.3** |
| Extension cord | 3 | 1.4 |
| Television table | 2 | 0.9 |
| Electrical cable | 2 | 0.9 |
| **Other** | **5** | **2.3** |
| Piece of fabric | 4 | 1.9 |
| Mop | 1 | 0.5 |
| **Missing** | **18** | **8.4** |
