# Supplementary material for: Characteristics of persons who died by suicide in prison in France: 2017–2018
Source: BMC Psychiatry. 2022 Jan 4;22:11. doi: 10.1186/s12888-021-03653-w (PMC8729083; doi:10.1186/s12888-021-03653-w)
Supplement: Supplementary file 4 — Additional file 4: Table 4 Suicide incidence rates according to some characteristics of the prisoners. [file 12888_2021_3653_MOESM4_ESM.docx]

Additional file 4

**Supplemental Table 4** Suicide incidence rates according to the characteristics of the prisoners

|  | Suicide cases  (n = 235) | | All prisoners  (n = 69155) | | Suicide rate / 10 000 PY^a^ | 95% CI^b^ | p value |
| --- | --- | --- | --- | --- | --- | --- | --- |
|  | n | % | n^c^ | % |  |  |  |
| Gender |  |  |  |  |  |  | 0.437 |
| *Female* | 12 | 5.1 | 2 397.4 | 3.5 | 25.0 | [10.9 – 39.2] |  |
| *Male* | 223 | 94.9 | 66 757.7 | 96.5 | 16.7 | [14.5 – 18.9] |  |
| Age (years) |  |  |  |  |  |  | < 0.001 |
| *<30* | 64 | 27.2 | 30 027.5 | 43.4 | 10.7 | [8.0 – 13.3] |  |
| *30-49* | 124 | 52.8 | 31 021.0 | 44.9 | 20.0 | [16.5 – 23.5] |  |
| ≥5*0* | 47 | 20.0 | 8 106.5 | 11.7 | 29.0 | [20.7 – 37.3] |  |
| Nationality |  |  |  |  |  |  | 0.294 |
| *French* | 192 | 81.7 | 53 604.0 | 77.5 | 17.9 | [15.4 – 20.4] |  |
| *Other* | 43 | 18.3 | 15 522.5 | 22.5 | 13.9 | [9.7 – 18] |  |
| Criminal status |  |  |  |  |  |  | <0.001 |
| *Remand status* | 114 | 48.5 | 19 885.4 | 71.3 | 28.7 | [10.1 – 14.5] |  |
| *Sentenced* | 121 | 51.5 | 49 322.7 | 28.7 | 12.3 | [23.4 – 33.9] |  |

^a^ Person-Years ^b^ Confidence Interval ^c^ Average of headcounts of the 1st of January 2017, the 1st of January 2018 and the 1st of January 2019
